# Genomic insights into extended-spectrum β-lactamase- and plasmid-borne AmpC-producing Escherichia coli transmission between humans and livestock in rural Cambodia

**DOI:** 10.1099/jmm.0.001988

**Published:** 2025-03-13

**Authors:** Ebraheem D. Elmarghani, John H.-O. Pettersson, Clara Atterby, Rachel A. Hickman, Sokerya Seng, Sorn San, Kristina Osbjer, Ulf Magnusson, Evangelos Mourkas, Josef D. Järhult

**Affiliations:** 1 Zoonosis Science Center, Department of Medical Sciences, Uppsala University, Uppsala, Sweden; 2 Clinical Microbiology and Hospital Hygiene, Uppsala University Hospital, Uppsala, Sweden; 3 Department of Microbiology and Immunology, Peter Doherty Institute for Infection and Immunity, University of Melbourne, Melbourne, Victoria, Australia; 4 Department of Microbiology, Swedish Veterinary Agency, Uppsala, Sweden; 5 Department of Internal Medicine, Visby Hospital, Visby, Sweden; 6 Food and Agriculture Organization of the United Nations, Phnom Penh, Cambodia; 7 General Directorate of Animal Health and Production, Phnom Penh, Cambodia; 8 Department of Clinical Sciences, Swedish University of Agricultural Sciences, Uppsala, Sweden; 9 International Centre for Antimicrobial Resistance Solutions, Copenhagen, Denmark; 10 Antimicrobial Research Unit, College of Health Sciences, University of KwaZulu-Natal, Durban, South Africa

**Keywords:** antimicrobial resistance, carbapenemase, extended spectrum beta-lactamase, genomics, horizontal gene transfer, livestock, One Health, plasmid-borne AmpC

## Abstract

**Introduction.** The global spread of extended-spectrum cephalosporinase-producing *Escherichia coli* (producing extended-spectrum *β*-lactamase or plasmid-borne AmpC, hereafter ESC-Ec) is a major public health concern. Whilst extensively studied in high-income countries, the transmission pathways between humans and animals in low- and middle-income countries (LMICs) remain unclear. In rural Cambodia, the asymptomatic carriage and transmission dynamics of ESC-Ec between humans and animals living in close proximity are poorly understood, highlighting the need for targeted research in this area.

**Gap statement.** An enhanced understanding of the genetic epidemiology of ESC-Ec can enable mitigation strategies to reduce the burden of disease and drug-resistant infections in LMIC settings.

**Aim.** This study aimed to investigate the genetic relatedness and genotypic antibiotic resistance profiles of ESC-Ec strains from humans and livestock in rural Cambodia and to identify patterns of antimicrobial resistance (AMR) gene transmission between hosts and across households and villages.

**Methodology.** Faecal samples were collected from 307 humans and 285 livestock in 100 households in or near Kampong Cham Province in rural Cambodia. From these samples, 108 ESC-Ec strains were subjected to whole-genome sequencing. Core genome MLST (cgMLST) and phylogenetic analysis determined genetic relationships between strains. All strains were screened for the presence of antibiotic resistance genes and plasmids.

**Results.** Human and livestock isolates were assigned to six phylogroups, with phylogroup A being the most common (56.5%). MLST identified 50 sequence types (STs), 17 of which were shared between humans and animals, with ST155 being the most prevalent. cgMLST revealed 97 distinct cgMLST sequence types (cgST), indicating strain sharing between humans and animals. Additionally, AMR gene analysis showed widespread resistance, with genes from the *bla*_CTX-M_ group detected in 84.2% of isolates. Notably, AMR genes such as *aph(3'')-Ib–sul2* co-occurred in 50% of isolates. Finally, plasmid analysis identified IncF plasmids in 75.9% of isolates, likely facilitating AMR gene transmission across hosts.

**Conclusions.** Our findings demonstrate that ESC-Ec strains and their AMR genes are transmitted between humans and livestock in rural Cambodia, likely driven by both clonal spread and plasmid-mediated horizontal gene transfer. These results highlight the urgent need for antimicrobial stewardship and infection control strategies to mitigate the spread of multidrug-resistant pathogens in both human and animal populations.

**Impact Statement**

This study highlights the widespread presence and transmission of extended-spectrum *β*-lactamase- and plasmid-borne AmpC-producing *Escherichia coli* between humans and animals in rural Cambodia. The high prevalence of multidrug-resistant strains poses a significant public health risk, emphasizing the need for enhanced surveillance and control measures to mitigate the spread of antimicrobial resistance in low-resource settings.

## Data Summary

Raw Illumina reads have been deposited to the GenBank database in the National Center for Biotechnology Information under the BioProject accession PRJNA1179385 (https://www.ncbi.nlm.nih.gov). SRA accession numbers and BioSample accessions for each genome are provided in Table S1 (available in the online Supplementary Material). The full set of assemblies has been deposited at the public data repository FigShare under https://doi.org/10.6084/m9.figshare.27323736.

The Microreact link for the genomic analysis is provided: https://microreact.org/project/x8s1jMA8HzmVNXRJ9WLEXB-cambodia-project.

## Introduction

*Escherichia coli* (*E. coli*) is a Gram-negative bacterium that is part of the normal microbiota in humans and animals [1,2]. This micro-organism has demonstrated the ability to acquire virulence determinants and/or antibiotic resistance genes (ARGs) from environmental sources, thereby enabling it to cause enteric or extraintestinal infections [2,3]. Furthermore, *E. coli* has been documented to undergo transmission between various hosts, facilitated by direct or indirect contact [2,4].

The overuse and misuse of antibiotics in human medicine, agriculture and animal husbandry have significantly contributed to the global antimicrobial resistance (AMR) crisis [5]. This has led to a rise in infections that are increasingly difficult to treat, in both humans and animals [6,7]. In response, the World Health Organization (WHO) has ranked antimicrobial drugs according to their importance in human medicine, with broad-spectrum cephalosporins and carbapenems listed among the highest priority [8]. The WHO has also highlighted the urgent need to address the growing threat posed by critical priority pathogens including *E. coli* [9,10]. Internationally spread high-risk *E. coli* clones, such as ST131 and ST410, have become major contributors to the spread of resistant infections [11,12]. Their genetic plasticity allows them to persist in various environments [13] and transfer resistance genes to other bacteria through mobile genetic elements (MGEs), promoting resistance to critical antibiotics, including colistin, carbapenems, cephalosporins and fluoroquinolones [14]. The global rise of extended-spectrum *β*-lactamase (ESBL)-producing *E. coli*, which confers resistance to third-generation cephalosporins, and their transmission between humans and animals has been documented before [3,15]. *E. coli* producing *β*-lactamases encoded by plasmid-borne AmpC (pAmpC) carry resistance to 3GC that can spread via horizontal gene transfer (HGT) and have been described as an understudied problem in Southeast Asia [16]. Together, these two types of resistant *E. coli* constitute an AMR problem complex and are hereafter referred to as extended-spectrum cephalosporinase-producing *E. coli* (ESC-Ec).

Genotypic methods, such as MLST and Clermont phylo-typing, have been widely used to classify *E. coli* strains and to study their clonal diversity and epidemiology [6,12, 17]. Several studies have inferred the sharing of clones between sources and transmission using phylogroups and/or MLST [18]. However, both methods rely on a limited number of genes providing an incomplete picture of transmission dynamics whilst also failing to capture accessory genome information, including ARGs. Whole-genome sequencing (WGS) offers a more comprehensive approach, enabling detailed phylogenetic analysis and identification of AGRs, as well as MGEs that facilitate the transfer of resistance genes.

The prevalence of antibiotic-resistant *E. coli* varies between low- and middle-income countries (LMICs) and high-income countries (HICs). In LMICs, factors like poor sanitation, limited access to clean water and inadequate healthcare infrastructure contribute to a higher burden of *E. coli* infections [19]. In many African countries, poultry serves as a major reservoir of ESBL-producing *E. coli* (ESBL-Ec), posing a risk to human health through the food chain and through direct contact with farmers [20]. In Kenya, a study revealed widespread antimicrobial-resistant *E. coli* among humans, livestock and wildlife, highlighting the importance of understanding interspecies transmission [21]. In our previous study in Cambodia, we frequently found ESC-Ec in faecal samples from women and young children potentially due to direct exposure to animal manure and slaughter products [22].

Understanding the prevalence of ESC-Ec in the gut of healthy humans requires an understanding of transmission dynamics between reservoirs. In this study, we used WGS to analyse ESC-Ec isolates collected from humans and livestock in rural Cambodia [22]. By characterizing cgMLST clusters and whole-genome phylogenies, we aimed to uncover transmission patterns in a geographically defined area. Additionally, we screened all genomes for ARGs and plasmids to investigate the role of HGT in the spread of resistance.

## Methods

### Sampling and data collection in rural Cambodia

Faecal samples were collected in ten households each in ten villages in or near Kampong Cham Province, Cambodia, in May 2011, as previously described [23,24]. Verbal consent was obtained from participating members of the household or their guardians before inclusion in the study. A total of 307 human and 285 livestock samples (113 ruminants, 138 poultry and 39 pigs) were obtained from 100 households in rural Cambodia. From these samples, 108 *E. coli* strains, displaying *in vitro* resistance to carbapenems and/or third-generation cephalosporins, were selected for WGS, consistent with the latest priority list from WHO [8,9].

### Culture, DNA extraction and sequencing

Isolates were cultured using three chromogenic media [CHROMagarC3GR (CHROMagar), chromID OXA-48 (bioMérieux) and chromID CARBA (bioMérieux)] to obtain antibiotic-resistant *E. coli,* as described before [22]. As part of the previous study [22], ESBL/pAmpC/carbapenemase production was confirmed for all isolates by detecting ESBL, pAmpC or OXA-48 genes through PCR and sequencing of the PCR products. Additionally, antibiotic susceptibility tests were performed using the EUCAST disc diffusion method and epidemiological cut-offs, as described before [22]. In the case of an ESBL phenotype, a double disc synergy test [25] was performed, and if indicative of ESBL production, the isolate was included for further analysis despite PCR results. Species was confirmed using MALDI-TOF MS, following previously described protocols [22,24]. Genomic DNA was extracted using the Norgen Biotek high-throughput bacterial genomic DNA isolation kit (SKU 17950) according to the manufacturer’s instructions. DNA concentration was quantified on a Nanodrop prior to normalization and sequencing. High-throughput sequencing (PE150bp) was performed on the Illumina platform by Novogene.

### Genome assembly, MLST and phylogroup classification

Isolates were labelled according to their ID numbers (C1–C112) and annotated with corresponding coordinates, including village number, household code, individual code and source (human or animal) (Table S1). Genomic analysis was performed using the QAssembly pipeline v3.61, integrated into Enterobase [26]. Briefly, Illumina raw reads were quality- and adapter-trimmed using Sickle v1.33 [27] with a quality threshold of 10 to trim bases with low scores [26]. Trimmed reads were assembled with SPAdes v3.9.1, using the –careful mode, which employs the BayesHammer error correction tool to minimize mismatches, and k-mer sizes of 21, 33, 55 and 77 [28]. BWA v0.7.12-r1039 was used to align reads back onto the assemblies to enhance the accuracy of consensus base calls [29]. Quality control of the assemblies was based on established criteria for the *Escherichia* genus, including genome size (3.7–6.4 Mbp), N50 value greater than 20 kb, number of contigs (≤800), proportion of scaffolding placeholders (N’s) below 3% and more than 70% of contigs assigned as *E. coli* using Kraken v0.10.5-beta [26,30]. Additional genome quality checks were performed with CheckM v1.1.3 [26,31], ensuring completeness above 99% and contamination below 1% (Table S1). Assemblies with insufficient coverage depth (<30×) were excluded. The assembled genomes were analysed to identify their MLST and cgMLST sequence types (cgST) using the NSer genotype integrated into the Enterobase database [26,32]. Additionally, all genomes were assigned to phylogroups using the Clermont phylo-typing method [26,33].

### Genome and phylogenetic analysis

Genomes were annotated using Prokka v1.11 [34], and assemblies were mapped to the reference genome of *E. coli* 169757 (Enterobase strain name: ESC_ZB5673AA_AS; NCBI accession number: JAAKGF000000000.1) using refMapper v1.0 [26]. A total of 32 037 967 SNPs were aligned to the reference genome with the LAST package v5.88 [26], using a minimum score of 100 bases for initial matches. The maf-convert feature was used to generate SNP alignments. SNPs with base quality scores below 10 or alignment ambiguities above 0.1 were excluded. After filtering out SNPs that are present in repeat regions or in regions that are missing in 10% or more of all the genomes using refMasker, the remaining 282 923 SNPs, present in more than 95% of all isolates, were combined into an SNP matrix file using refMapperMatrix v2.0 [26]. The resulting matrix was used to generate a phylogenetic tree with the maximum-likelihood algorithm implemented in RAxML v8.2.9 [35,36], using the GTRGAMMA substitution model and 100 bootstrap replicates to assess branch support.

### Screening for ARGs and plasmids

All genomes were screened for the presence of ARGs using ResFinder v4.6.1 [37,38]. A positive hit was considered when sequences matched with >90% identity over >90% of the sequence length. The presence of plasmids was investigated using PlasmidFinder v2.0.1 [38,39]. A positive hit was considered when the sequence matched with >90% identity over >70% of the sequence length. The association between AMR phenotype and genotype was tested using chi-square and Fisher’s exact tests. Results were considered statistically significant when *P*<0.05.

## Results

### Genomic comparison of *E. coli* isolated from human and livestock carriage

**Fig. 1.**
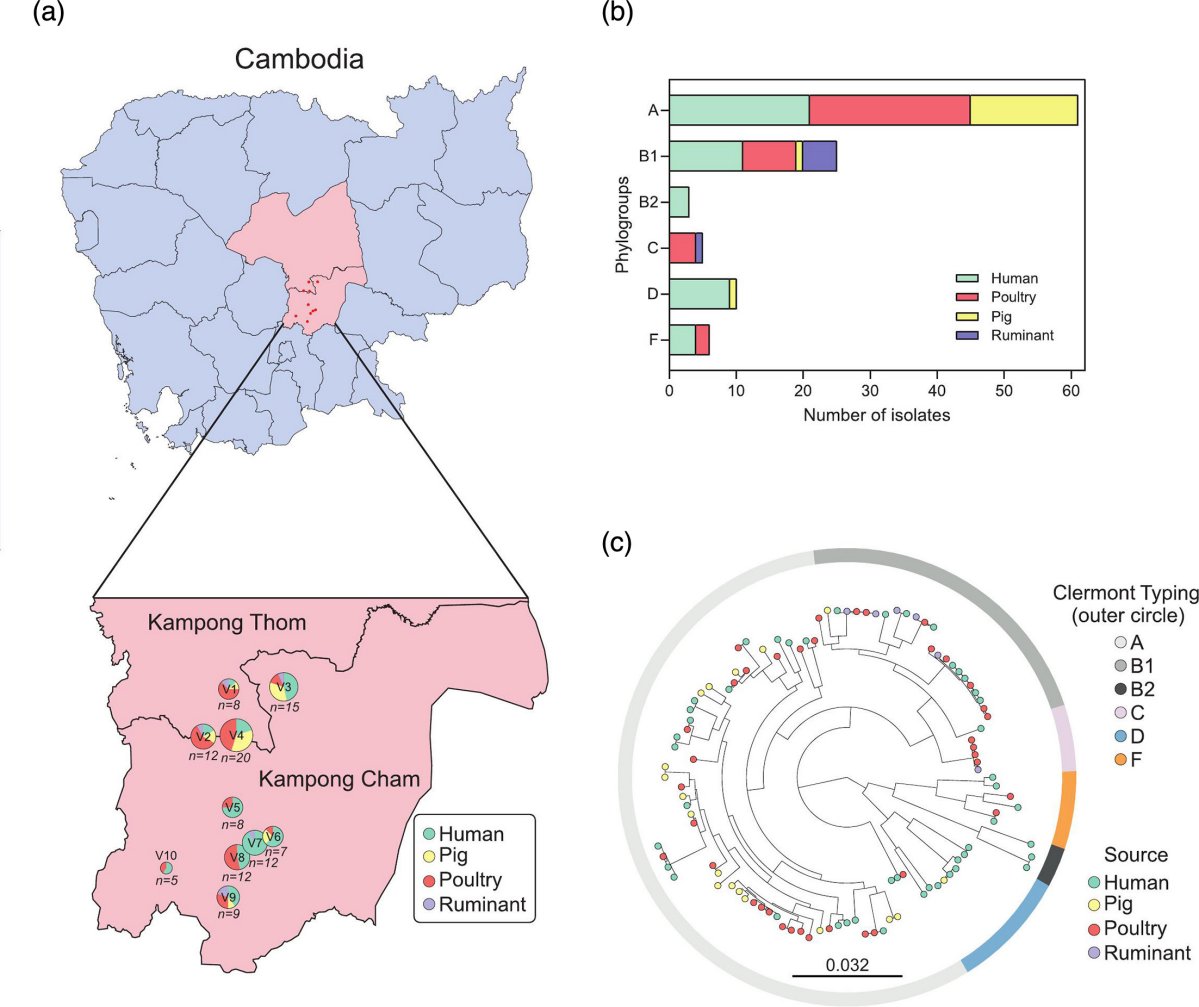
(a) Map of Cambodia showing the village locations (V1–V10) highlighted by red dots. The zoom-in view of the sampling collection sites shows the distribution of ESBL-Ec strains from humans and livestock. (b) Distribution of human and livestock samples within the six known phylogroups. The *y*-axis highlights the six known phylogroups, whilst the *x*-axis shows the number of isolates from humans (green), pigs (yellow), poultry (red) and ruminants (violet). (c) Population structure of 108 ESC-Ec isolates. A maximum-likelihood phylogeny was reconstructed with RAxML using the GTRGAMMA model based on 282 923 core SNPs. The scale bar indicates the estimated number of substitutions per site. Leaves are coloured by host including isolates from humans (green), pigs (yellow), poultry (red) and ruminants (violet). The inferred phylogroups based on Clermont typing are annotated in the outer circle.

Faecal samples were collected in May 2011 from 100 households across ten villages in rural Cambodia (Fig. 1a). A total of 108 ESC-Ec isolates were obtained from healthy humans (*n*=46), poultry (*n*=38; chicken and domesticated ducks), pigs (*n*=18) and ruminants (*n*=6; cattle and buffalo) (Table S1). The genomes clustered into six known *E. coli* phylogroups (A, B1, B2, C, D and F) based on Clermont phylo-typing (Fig. 1b). The predominant group was A (61 out of 108; 56.5%), followed by B1 (24 out of 108; 22.2%), D (9 out of 108; 8.3%), F (6 out of 108; 5.5%), C (5 out of 108; 4.6%) and B2, representing the smallest group (3 out of 108; 2.7%) (Fig. 1b). Based on MLST, the 108 strains were classified into 50 STs, with three genomes assigned a novel sequence type (ST) (ST15565) (Table S1). Seventeen STs were found in both humans and animals, with ST155 being the most prevalent (11 out of 108; 10.2%), isolated from poultry (*n*=5), humans (*n*=5) and a ruminant (*n*=1) (Table S1). Seven STs were shared among isolates from a single host, including ST131 in two human isolates, ST44 in two pig isolates and ST6782 in two poultry isolates. The remaining 26 STs were assigned to 26 distinct isolates, each found in a single host species (Fig. S1A, Table S1).

### cgST groups shared between humans and animals across households and villages

**Fig. 2.**
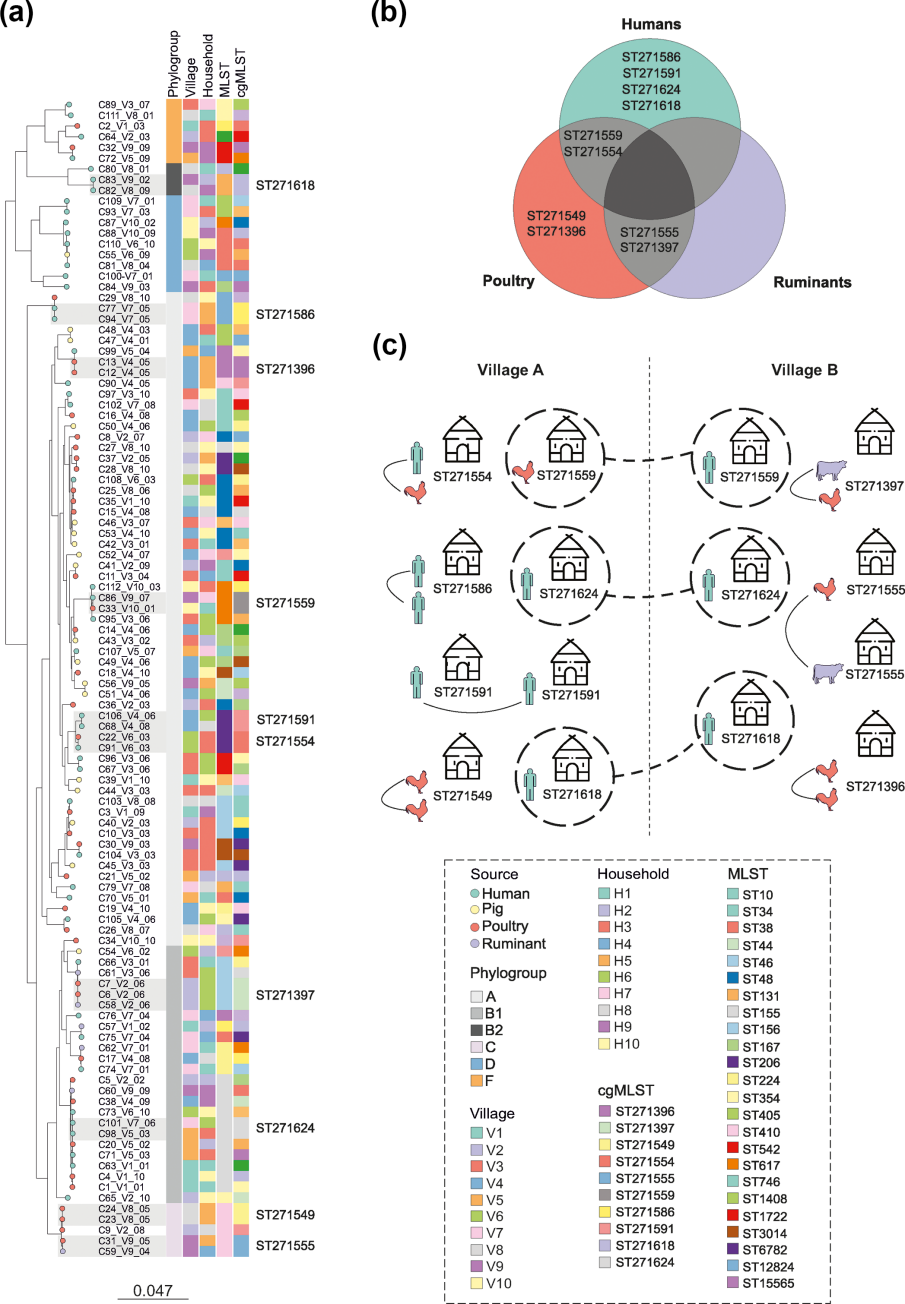
(a) A maximum-likelihood phylogeny reconstructed for 108 ESC-Ec isolates using 282 923 core SNPs and the GTRGAMMA substitution model implemented in RAxML. Common STs, based on MLST and cgMLST, as well as phylogroups, based on Clermont phylo-typing, village and household metadata are indicated as coloured blocks next to the phylogenetic tree. The origin of each isolate is shown in the leaves for humans (green), pigs (yellow), poultry (red) and ruminants (violet). The leaf names correspond to sample metadata information including the ID of the sample (Cx), village number (V1–V10) and household number (01–10). Village and household metadata are also indicated as blocks next to the phylogeny. The ten highly similar cgSTs circulating among humans and livestock across households and villages are shaded in grey, with the cgST annotated. The scale bar indicates the estimated number of substitutions per site. (b) Venn diagram displaying the ten highly similar cgSTs shared between humans and livestock animals. (c) Illustration showing the distribution of the ten cgSTs, which are shared among humans and animals within and between households and villages.

To better understand genetic relationships and potential transmission routes within and between households and villages, we used cgST for analysis. Compared to MLST, cgST captures more genomic variation, providing greater resolution of strain relatedness. The 108 *E. coli* genomes from humans and animals were grouped into 97 distinct cgSTs, with ten cgSTs found in more than one isolate (Fig. 2a, Table S1). Among these, four cgSTs (ST271586, ST271591, ST271618 and ST271624) were found only in human isolates, with ST271586 shared within the same household (Fig. 2b, c), whilst the others were detected in different households within the same or different villages (Figs 2b, c and S1B). Two cgSTs (ST271396 and ST271549) were isolated exclusively from poultry within the same household (Figs 2b, c and S1B). The remaining four cgSTs were isolated from multiple sources: ST271554 and ST271397 were isolated from humans and poultry and poultry and ruminants, in the same household, respectively (Fig. 2b, c). ST271555 and ST271559 were shared between ruminants and poultry and humans and poultry, across different households in the same or different villages (Figs 2b, c and S1B). These findings demonstrate that highly similar ESC-Ec strains are asymptomatically carried by both humans and animals, indicating clonal transmission occurring within and between households and villages, including zoonotic transmission.

### Widespread AMR gene distribution in ESC-Ec strains from humans and animals

All isolates were selected based on their resistance to cephalosporins and/or carbapenems, and antimicrobial susceptibility was determined using disc diffusion assays of seven antibiotics: ciprofloxacin, chloramphenicol, gentamicin, meropenem, tetracycline, sulphamethoxazole/trimethoprim and piperacillin/tazobactam (Table S2), as described before [22]. To obtain a comprehensive resistance profile, we screened the genomes for the presence of AMR genes using ResFinder [37,38]. We identified a total of 63 AMR genes conferring predicted resistance to various antibiotics, including beta-lactams, tetracyclines, sulphonamides, trimethoprim, aminoglycosides, amphenicol, fluoroquinolones, lincosamides, macrolides and colistin (Table S2). Concordance between putative resistance genotypes and laboratory phenotypes was observed for all antibiotics tested except for ciprofloxacin (Table S3) as previously described [40].

ESBL genes, in the form of *bla*_CTX-M_, were found in 84.2% (91 out of 108) of isolates, including humans (43 out of 46; 93.4%), pigs (15 out of 18; 83.3%), poultry (29 out of 38; 76.3%) and ruminants (4 out of 6; 66.7%) (Table 1). The most common CTX-M subtypes were CTX-M-55 (32 out of 108, 29.6%), CTX-M-27 (31 out of 108, 28.7%), CTX-M-14 (17 out of 108, 15.7%) and CTX-M-15 (10 out of 108, 9.2%) (Table S2). We confirmed the finding of *bla*_OXA-48_ in one out of two isolates from humans reported previously in a PCR-based study of the same samples (Table S2) [22]; the other *bla*_OXA-48_-carrying isolate could unfortunately not be included in the present study due to poor sequence quality. Tetracycline resistance was common, primarily driven by the *tetA* gene, which was found in 66.7% (72 out of 108) of isolates, including humans (32 out of 46; 69.6%), poultry (25 out of 38; 65.8%), pigs (12 out of 18; 66.7%) and ruminants (3 out of 6; 50.0%). Sulphonamide resistance was also common, primarily conferred by *sul2*, which was detected in 64.8% (70 out of 108) of isolates, including humans (32 out of 46; 69.6%), poultry (22 out of 38; 57.9%), pigs (14 out of 18; 77.8%) and ruminants (2 out of 6; 33.3%) (Table 1). Among aminoglycoside resistance genes, *aph(3'')-Ib* was the most common, found in 54.6% (59 out of 108) of the isolates, including humans (27 out of 46; 58.7%), poultry (18 out of 38; 47.4%), pigs (12 out of 18; 66.7%) and ruminants (2 out of 6; 33.3%) (Table 1). The gene *aac(6')-Ib-cr*, which confers combined resistance to aminoglycosides and quinolones [41], was present in 8.3% (9 out of 108) of isolates, including humans (4 out of 46 : 8.7%), poultry (3 out of 38; 7.9%), ruminants (1 out of 6; 16.7%) and pigs (1 out of 18; 5.5%) (Table 1). Nearly all of the isolates (106 out of 108; 98.1%) were multidrug-resistant (MDR), whilst one isolate (1 out of 108; 0.9%) was classified as extensive drug-resistant based on *in silico* predicted resistance (Table S4). Our results show the widespread distribution of AMR genes in ESC-Ec strains across humans and animals, highlighting the risk posed by MDR strains circulating between various species.

**Table 1.** Distribution of most common ARGs in ESC-Ec isolates from humans and animals

Antibiotic class	Major AMR gene group	No. of isolates (%)				Total gene no.
		Humans (*n*=46)	Poultry (*n*=38)	Pigs (*n*=18)	Ruminants (*n*=6)	
Beta-lactams	*bla*_CTX-M_	43 (93.5)	29 (76.3)	15 (83.3)	4 (66.7)	91
	*bla*_TEM_	34 (73.9)	28 (73.9)	12 (66.7)	5 (83.3)	79
Tetracyclines	*tet(A)*	32 (69.6)	25 (65.8)	12 (66.7)	3 (50.0)	72
Sulphonamides	*sul2*	32 (69.6)	22 (57.9)	14 (77.8)	2 (33.3)	70
Trimethoprim	*dfrA14*	20 (43.5)	19 (50)	7 (38.9)	2 (33.3)	48
Aminoglycosides	*aph(3’’)-Ib*	27 (58.7)	18 (47.4)	12 (66.7)	2 (33.3)	59
	*aadA2*	15 (32.6)	13 (34.2)	7 (38.9)	1 (16.7)	36
	*aac(3)-IId*	8 (17.4)	6 (15.8)	4 (22.2)	1 (16.7)	19
	*ant(2’’)-Ia*	1 (2.2)	2 (5.3)	0	0	3
Amphenicol	*catA*	20 (43.5)	14 (36.9)	11 (61.1)	3 (50)	48
	*cmlA1*	7 (15.2)	6 (15.8)	5 (27.8)	1 (16.7)	19
	*floR*	8 (17.4)	0	3 (16.7)	0	11
Fluoroquinolones	*qnrS1*	17 (37)	19 (50)	8 (44.4)	1 (16.7)	45
	*qepA1*	2 (4.3)	1 (2.6)	0	0	3
Lincosamides	*lnu(F)*	10 (21.7)	9 (23.7)	4 (22.2)	0	23
Macrolides	*mph(A)*	24 (52.2)	12 (31.6)	8 (44.4)	2 (33.3)	46
Colistin	*mcr-1.1*	1 (2.2)	3 (7.9)	3 (16.7)	0	7
Aminoglycoside-quinolones	*aac(6’)-Ib-cr*	4 (8.7)	3 (7.9)	1 (5.5)	1 (16.7)	9

To investigate the presence of plasmids in our genome collection and their potential role in harbouring AMR genes, we screened our sequences using PlasmidFinder [38,39]. A total of 227 plasmids, classified into 11 families, were identified (Table S5). The IncF family was the most prevalent, detected in 75.9% (82 out of 108) of isolates, followed by Col (36.1%; 39 out of 108), p0111 (25%; 27 out of 108), IncX (21.3%; 23 out of 108), IncH (16.9%; 20 out of 108) and IncQ1 (11.1%; 12 out of 108) (Table S5). Other plasmid families were less common (each detected in less than 10% of the isolates) (Table S5).

### Genetic associations in ESC-Ec suggest HGT across lineages and hosts

**Fig. 3.**
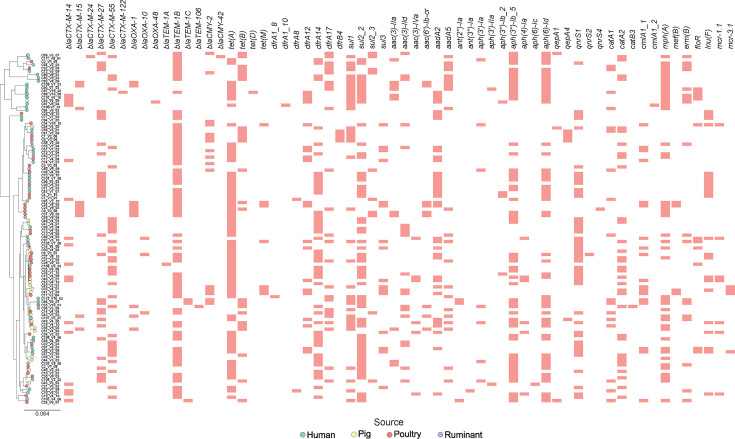
Presence of ARGs in ESC-Ec sampled from human and livestock carriage. The same phylogenetic tree was used with the leaves coloured by host including isolates from humans (green), pigs (yellow), poultry (red) and ruminants (violet). The presence of all ARGs identified using ResFinder is indicated by red-coloured blocks. The scale bar indicates the estimated number of substitutions per site.

AMR genes are often found in close proximity within the genome, forming clusters [42,43]. For example, ESBL genes frequently co-occur with quinolone resistance genes (*qnr*) [44,45] and sulphonamide resistance genes (*sul1*) [45]. In our study, 97.2% (105 out of 108) of isolates showed at least two AMR genes co-occurring on the same contig, resulting in 64 distinct genetic associations (Table S6). The main four of these associations were present in 74.1% (80 out of 108) of the isolates (Table S6). The most common association, mainly formed by *aph(3’’)-Ib–sul2*, was detected in 50% (54 out of 108) of isolates, including humans (25 out of 46; 54.3%), poultry (16 out of 38; 42.1%), pigs (11 out of 18; 61.1%) and ruminants (2 out of 6; 33.3%) (Table S6). The second most common association, mainly formed by *sul1–dfrA17/12–aadA2/5*, was found in 24.1% (26 out of 108) of isolates, including humans (13 out of 46; 28.3%), poultry (6 out of 38; 15.8%) and pigs (7 out of 18; 38.9%), whilst the third most common association formed by *aadA2–lnu(F)* was detected in 15.7% (17 out of 108) of isolates, including humans (8 out of 46; 17.4%), poultry (8 out of 38; 21%) and a pig (1 out of 18; 5.5%) (Table S6). The fourth association (*bla*_CTX-M-55_–*qnrS1*) was detected in 13% (14 out of 108) of isolates, including humans (4 out of 46; 8.7%), poultry (7 out of 38; 18.4%) and pigs (2 out of 18; 11.1%). Additionally, more than one genetic association was found in different contigs within the same genome for 62 different isolates (Table S6), highlighting the potential for HGT mechanisms that involve combinations of genetic clusters moving within the genome (Fig. 3).

## Discussion

The rising prevalence of ESBL-Ec in human infections is a significant global health concern [46,49]. Whilst reservoirs and transmission routes of whole genome-sequenced ESBL-Ec have been studied in HICs [50,52], limited information is available for LMICs [53]. In this study, we investigated the prevalence, genetic relatedness and AMR of ESC-Ec (including also the less-studied pAmpC) in humans and livestock living in the same or different households in ten villages in rural Cambodia.

The majority of our isolates (78.7%) belonged to phylogroups A and B1, commonly associated with commensal strains in both humans and livestock [54]. Phylogroup B2, often linked to human invasive disease [54,55], was not found in any of the livestock samples, supporting the idea that livestock are not a direct source of B2-related infections [4]. Conversely, phylogroup C was exclusively found in livestock, whilst phylogroups A, D and F included strains from both humans and livestock, indicating that ESC-Ec strains from different phylogroups are carried asymptomatically by multiple hosts [52]. Genome comparison based on MLST has shown that certain ESC-Ec STs are shared between humans and livestock [56,60]. We confirmed these findings for ST10, ST38, ST46, ST48, ST155, ST156, ST206, ST224, ST410, ST617 and ST746, suggesting that strains circulate between humans and livestock in the same or different households and villages in rural Cambodia. Meat and fish have been proposed as a source of human ESC-Ec carriage in Cambodia [61], but to the best of our knowledge, this is the first study that employs WGS to investigate the genetic epidemiology of ESC-Ec carriage in humans and livestock in rural Cambodia.

We used cgMLST instead of MLST to describe the sharing of genetically similar strains because it offers greater resolution in differentiating closely related isolates [62]. We identified ten cgSTs with at least two isolates circulating across the same and different households and villages (Fig. 2). Some of these cgSTs were shared between humans, animals or both, aligning with previous studies that demonstrated human-to-human transmission [63], and transmission between animals and humans [64,65]. Additionally, we observed cgST sharing among humans, animals and between humans and animals living in the same household. Sharing was also noted between different households in the same village and different villages, consistent with inter-household and inter-host transmission patterns reported in Kenya [66]. These findings provide evidence of recent clonal transmission between humans and livestock, shaped by both household proximity and host type.

Our resistome analysis detected the presence of ESBL genes in ESC-Ec strains previously confirmed by PCR-based methods [22], predominantly from the *bla*_CTX-M_ and *bla*_TEM_ families, which are the most commonly reported genes globally [67,71]. Among the CTX-M subtypes, CTX-M-55, CTX-M-27, CTX-M-14 and CTX-M-15 were the most frequent, suggesting the diverse but prevalent nature of these subtypes in rural Cambodia, a trend also observed in India, Uganda and Cameroon [72,74]. These genes have also been found in ESBL-Ec from blood cultures in Cambodia, indicating their clinical importance [61,75].

Widespread resistance to other antibiotics, such as tetracycline, trimethoprim and sulphonamide, was also detected, with resistance genes *tet*, *dfr* and *sul* each present in more than 85% of all isolates, consistent with international findings [63,76,78]. Resistance to gentamycin, commonly used as a prophylactic in livestock in LMICs [79], was confirmed by the presence of the *aph(3’)-Ib* gene in nearly half of our isolates, aligning with a study from South Africa indicating frequent aminoglycoside resistance in ESBL-Ec strains [80]. A large proportion of our isolates exhibited *in silico* resistance against other antibiotic classes, with nearly all (98.1%) being MDR, consistent with high resistance rates seen in many first-line antibiotics in other LMICs [81,84]. *In silico* resistance was compared to antibiotic susceptibility testing data from our previous study [22], and we observed significant concordance for all seven tested antibiotics except for ciprofloxacin, consistent with the fact that ciprofloxacin resistance can occur also by point mutations in the chromosomal DNA and that the presence of a single ciprofloxacin-related ARG does not always result in phenotypic resistance.

The high prevalence of IncF plasmids in our ESC-Ec isolates, carrying *bla*_CTX-M_, aminoglycosides and quinolone resistance genes, suggests that plasmid-driven transmission may contribute to MDR. This is consistent with findings from others showing IncF plasmids as major vehicles for ARG transmission [69,70]. The frequent detection of Col plasmids carrying quinolone-resistant genes like *qnr* further supports the hypothesis that specific plasmid families may play a role in spreading resistance across bacterial communities [36]. These plasmid-mediated mechanisms underscore the urgent need for surveillance strategies targeting plasmid movement to mitigate the spread of MDR strains in both human and animal populations.

The mere ubiquitous distribution of AMR genes does not provide sufficient evidence for recent transfer of genes between reservoirs. To address this, we hypothesized that recent sharing would be mirrored in genes forming clusters co-localized on the same contig, likely mediated by MGEs (i.e. plasmids), as previously suggested for ESBL and other resistance genes [85]. There were multiple gene clusters containing genes that confer resistance to more than one antibiotic class (except for beta-lactams) as previously reported [86,88]. The findings were consistent with distinct genetic clusters associated with either humans or livestock, as well as ones found in both [4]. For example, gene clusters solely consisting of *aph(3'')-Ib–sul2* appear in isolates from livestock, whilst gene cluster *bla*_CTX-M-55/27_–*qnrS1* was detected in isolates from multiple sources. Additionally, the common co-occurrence of multiple distinct gene clusters within a single genome suggests possible functional relatedness or evolutionary preservation [89]. Our results highlight the significant role of co-occurring ARGs in the transmission of resistance between humans and animals across households and villages. The co-occurrence of genes such as *bla*_CTX-M_ and *qnr* was commonly detected in strains shared between humans and poultry, reflecting the potential for AMR transmission through direct or indirect contact.

The widespread co-occurrence of ARGs, such as *aac(6')-Ib-cr* with bla_OXA_ and resistance genes for tetracycline and sulphonamides, suggests that habitual antibiotic use in both human and veterinary medicine is contributing to the persistence of MDR strains. In Cambodia, unregulated sales and inappropriate use of antibiotics in both humans and livestock intensify AMR selection pressure [90,92]. Antibiotics, such as colistin, gentamicin and enrofloxacin, are widely used in livestock for treatment, prophylaxis and growth promotion without veterinary oversight [79,93]. These practices promote the persistence of resistant strains and facilitate the exchange of resistance genes between human and animal reservoirs [22]. This co-selection of resistance genes not only complicates treatment options but also poses significant risks for public health, as highlighted by the detection of chloramphenicol resistance genes [84]. However, the relatively small sample size of human and poultry isolates is a limitation of this study. Additionally, the role of environmental contamination in AMR transmission was not addressed in this study and warrants future investigation as this is an important factor within the One Health framework. Similarly, a comprehensive analysis of plasmid transmission would require long-read sequencing or PCR-based replicon typing. Despite these limitations, the study still provides valuable insights into the transmission dynamics of ESC-Ec and highlights the role of HGT, likely through plasmids [2], in driving the spread of AMR genes between humans and animals in various settings.

## Conclusion

In this study, we investigate the role of both human and animal reservoirs in the transmission of ESC-Ec and associated AMR genes in rural Cambodia. We provide evidence for both clonal transmission and HGT between these reservoirs, complementing and strengthening our previous findings in Cambodia. The detection of ARGs sharing across households and villages suggests that interventions must extend beyond individual households to address community and regional transmission. The widespread prevalence of MDR strains highlights the urgent need for antimicrobial stewardship and infection control measures using a One Health approach, particularly in low-resource settings where human-animal interactions are frequent. By targeting both human and animal populations, such strategies could help limit the spread of resistant pathogens and reduce the public health burden of AMR.

## supplementary material

10.1099/jmm.0.001988 Table S1.

10.1099/jmm.0.001988 Table S2.

10.1099/jmm.0.001988 Table S3.

10.1099/jmm.0.001988 Table S4.

10.1099/jmm.0.001988 Table S5.

10.1099/jmm.0.001988 Table S6.

10.1099/jmm.0.001988 Supplementary Material 1.

## Abbreviations

AMR: antimicrobial resistance
ARGs: antibiotic resistance genes
cgMLST: core genome MLST
cgST: cgMLST sequence types
ESBL: extended-spectrum *β*-lactamase
ESBL-Ec: ESBL-producing *E. coli*
ESC-Ec: extended-spectrum cephalosporinase-producing *Escherichia coli*
HGT: horizontal gene transfer
HICs: high-income countries
LMICs: low- and middle-income countries
MDR: multidrug-resistant
MGEs: mobile genetic elements
pAmpC: plasmid-borne AmpC
STs: sequence types
WGS: whole-genome sequencing
WHO: World Health Organization
