# Supplementary material for: Genomic insights into extended-spectrum β-lactamase- and plasmid-borne AmpC-producing Escherichia coli transmission between humans and livestock in rural Cambodia
Source: J Med Microbiol. 2025 Mar 13;74(3):001988. doi: 10.1099/jmm.0.001988 (PMC11915462; doi:10.1099/jmm.0.001988)
Supplement: Supplementary Material 1. [file jmm-74-01988-s007.pdf]

Supplementary Information for

Genomic insights into ESBL- and pAmpC-producing *Escherichia coli* transmission between humans and livestock in rural Cambodia

Ebraheem D. Elmarghani[1], John H.-O. Pettersson[1,2,3], Clara Atterby[1,4], Rachel A. Hickman[1,2], Sokerya Seng[5], Sorn San[6], Kristina Osbjer[7,8#,9#] Ulf Magnusson[7], Evangelos Mourkas[1*], Josef D. Järhult[1*]

[1] Zoonosis Science Center, Department of Medical Sciences, Uppsala University, Uppsala, Sweden;

[2] Clinical Microbiology and Hospital Hygiene, Uppsala University Hospital, Uppsala, Sweden;

[3] Department of Microbiology and Immunology, Peter Doherty Institute for Infection and Immunity, University of Melbourne, Melbourne, Victoria, Australia;

[4] Department of Internal Medicine, Visby Hospital, Visby, Sweden;

[5] Food and Agriculture Organization of the United Nations, Phnom Penh, Cambodia;

[6] General Directorate of Animal Health and Production, Phnom Penh, Cambodia;

[7] Department of Clinical Sciences, Swedish University of Agricultural Sciences, Uppsala, Sweden

[8] International Centre for Antimicrobial Resistance Solutions, Copenhagen, Denmark

[9] Antimicrobial Research Unit, College of Health Sciences, University of KwaZulu-Natal, Durban, South Africa

[#] Current workplace

This file includes

Figures legend and Figure S1
Legends for Tables S1 to S6

Supplementary figures

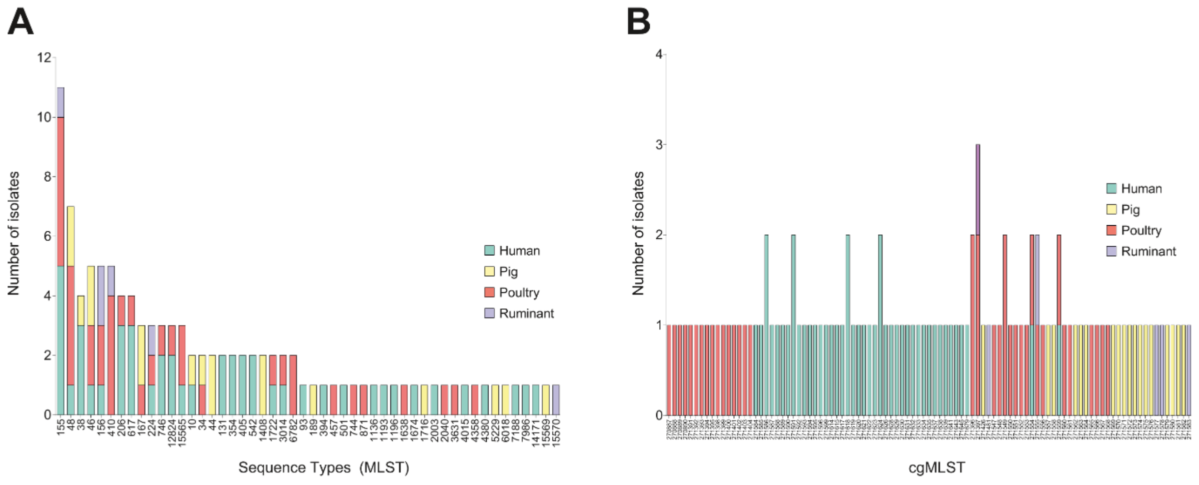

Supplementary figure 1. MLST and cgMLST distribution in ESBL-Ec isolates from humans and livestock (**A**)**.** A total of 50 STs were identified from humans and animals based on MLST scheme for all ESBL-Ec. The *x*-axis shows the distribution of different STs among humans (green), pigs (yellow), poultry (red) and ruminants (purple). (**B**) A total of 97 cgSTs were identified in humans and livestock based on cgMLST. The *x*-axis shows the distribution of different cgSTs among humans (green), pigs (yellow), poultry (red) and ruminants (purple).

Supplementary table legends

Table S1. Isolate information for the genomes used in this study.

Table S2. Putative antibiotic resistance genes and AST data for all isolates used in this study.

Table S3. Concordance of AMR phenotypes and genotypes. The presence of putative genotypic resistance (G+) and number of phenotypically non-susceptible isolates (P+) is indicated.

Table S4. Classification of non-multidrug resistant, MDR and XDR in isolates from humans and livestock.

Table S5. Plasmid distribution in ESC-Ec isolates from humans and livestock.

Table S6. Co-occurrence of AMR genes detected in all genomes in this study.
